# Clinical acceptance of advanced visualization methods: a comparison study of 3D-print, virtual reality glasses, and 3D-display

**DOI:** 10.1186/s41205-022-00133-z

**Published:** 2022-01-30

**Authors:** Julian Louis Muff, Tobias Heye, Florian Markus Thieringer, Philipp Brantner

**Affiliations:** 1 Department of Radiology and Nuclear Medicine, University Hospital Basel, Basel, Switzerland; 2 Department of Oral and Cranio-Maxillofacial Surgery and 3D Print Lab, University Hospital Basel, Basel, Switzerland; 3 Department of Biomedical Engineering, University Hospital Basel, Basel, Switzerland; 4 Department of Radiology, Gesundheitszentrum Fricktal, Rheinfelden, Switzerland

**Keywords:** - 3D-print, - VR-glasses, - 3D-display, - 3D-screen, - three dimensional, - education

## Abstract

**Background:**

To compare different methods of three-dimensional representations, namely 3D-Print, Virtual Reality (VR)-Glasses and 3D-Display regarding the understanding of the pathology, accuracy of details, quality of the anatomical representation and technical operability and assessment of possible change in treatment in different disciplines and levels of professional experience.

**Methods:**

Interviews were conducted with twenty physicians from the disciplines of cardiology, oral and maxillofacial surgery, orthopedic surgery, and radiology between 2018 and 2020 at the University Hospital of Basel. They were all presented with three different three-dimensional clinical cases derived from CT data from their area of expertise, one case for each method. During this, the physicians were asked for their feedback written down on a pencil and paper questionnaire.

**Results:**

Concerning the understanding of the pathology and quality of the anatomical representation, VR-Glasses were rated best in three out of four disciplines and two out of three levels of professional experience. Regarding the accuracy of details, 3D-Display was rated best in three out of four disciplines and all levels of professional experience. As to operability, 3D-Display was consistently rated best in all levels of professional experience and all disciplines. Possible change in treatment was reported using 3D-Print in 33%, VR-Glasses in 44%, and 3D-Display in 33% of participants. Physicians with a professional experience of more than ten years reported no change in treatment using any method.

**Conclusions:**

3D-Print, VR-Glasses, and 3D-Displays are very well accepted, and a relevant percentage of participants with less than ten years of professional work experience could imagine a possible change in treatment using any of these three-dimensional methods. Our findings challenge scientists, technicians, and physicians to further develop these methods to improve the three-dimensional understanding of pathologies and to add value to the education of young and inexperienced physicians.

## Background

Medical imaging plays a paramount role in diagnosis, particularly volumetric computed tomography (CT) and magnetic resonance imaging (MRI) with isotropic voxels [1, 2]. Many diagnoses are made not only with interpretation of axial slices, but include medical intuition gained from 2D-Displays of volumetric data sets such as multiplanar reformatted images, maximum intensity projections, and 3D volume rendering. These are collectively defined as 3D visualizations [3]. Displays using traditional flat screens do not include information of depth [4]. For example, volume rendering has made three-dimensional visualization possible, however, adding an accurate spatial impression remains challenging.

Today’s most important available options to truly convey three-dimensional images are 3D-Printing, Virtual Reality (VR)-Glasses, and 3D-Displays. First, 3D-Printing has lately received growing attention from research [5]. Using the term “3D printing” for a search in Pubmed.gov resulted in six publications in 2000, 64 publications in 2010, and astonishing 3569 results in 2020 [5, 6]. Areas of application include anatomical models, surgical guides, prosthesis, implantable or non-implantable medical devices, printing patient-specific anatomy for visualization, micro-needle, bioprinting, and pharmacoprinting [7–18]. In contrast to other technologies, segmentation of the desired structure out of the initial dataset is necessary. Several groups of specific 3D-Printing techniques exist, comprising vat photopolymerization, material jetting, binder jetting, material extrusion, powder bed fusion, sheet lamination, and directed energy deposition [3]. Often used methods for printing are material extrusion (MEX) and vat photopolymerization (VP) [19, 20]. The most economical way is MEX. For MEX, a spool of filament such as polylactic acid (PLA) or acrylonitrile butadiene styrene (ABS) is fed through a heated nozzle [21]. The nozzle then moves along pre-programmed coordinates, extruding a layer of melted material on the build plate. Layer by layer is added to create a three-dimensional model. The second mode of presentation are VR-Glasses. VR is a computer-generated simulation of a three-dimensional environment with which a person can interact using special electronic equipment. This can be combined with a wearable headset called VR-Glasses. To achieve the effect of three-dimensionality, two screens inside the VR-Glasses show two images slightly shifted in relation to each other. This results in a stereoscopic impression similar to natural viewing, where stereoscopic viewing is achieved by perceiving images through the slightly different positions of our left and right eye. In medicine, the use of VR-Glasses has been reported in three-dimensional planning of surgeries, teaching and training, non-pharmacological pain management, and in-home therapy intervention for patients with stroke [22–32]. Only recently, VR-Glasses have become more employed in diagnostics [33, 34].

The third method, a 3D-Display, creates a realistic three-dimensional impression in contrast to the already existing three-dimensional renderings on a flat-screen. 3D-Displays use stereoscopic vision, where slightly different images are sent to each eye, leading to depth perception. There are two ways to realize this. An autostereoscopic 3D-Display works as a stand-alone screen. The second possibility are stereoscopic displays with the additional wearing of special glasses. Three-dimensional displays have seldom been used in medicine due to their higher costs compared to a normal 2D-Display and the occasional experience of visual discomfort [35]. Some applications in displaying 3D ultrasound data or intracranial MR angiography have been reported [36–38]. Lastly, augmented reality (AR) receives growing interest. AR is a blend of projected computer-generated images in a real environment e.g., in an operating theater [39]. However, AR was not assessed in this study.

3D-Printing, VR-Glasses, and 3D-Displays can enhance physicians’ understanding of pathology. However, research on the subject has mostly been restricted to limited comparison of these modalities. To our knowledge, no studies are comparing the three mentioned methods. Much uncertainty still exists about the application in different disciplines and how well received the three-dimensional representations methods are in various levels of professional experience. Furthermore, existing accounts fail to investigate if physicians state a possible change in treatment using the mentioned three-dimensional methods.

To address these research gaps, this study aims:

1. To compare the three mentioned methods of 3D representations by evaluating the understanding of the pathology, accuracy of details, quality of the anatomical representation, and technical operability and investigating how well received they are in different disciplines and different levels of professional experience.

2. To ascertain if physicians state a possible change in treatment using 3D-Print, VR-Glasses, or a 3D-Display.

## Methods

### Participants

Interviews were conducted with twenty-three physicians employed at the University Hospital of Basel, Switzerland. Participants were recruited from disciplines where a potential for three-dimensional representation was identified, namely cardiology, oral and maxillofacial surgery, orthopedic surgery, and radiology. The possible participants were contacted via e-mail or asked personally to participate in this study. In total, fifty-two individuals were contacted. The physicians who found the time and were willing to attend a meeting in the 3D-PrintLab were included in this study. Participation was voluntary, and everyone agreed to the use of their data. In the end, twenty-three interviews were conducted between December 2018 and March 2020. Two participants (one student and one technical adviser) failed to comply with the selection criteria (not being a physician) and were therefore excluded. One physician had to be excluded due to technical problems in demonstrating the case on the VR-Glasses. The final cohort of this study consisted of twenty participants. Five physicians from each of the following disciplines took part: Cardiology, oral and maxillofacial surgery, orthopedic surgery, and radiology. Five participants had ≤5 years of professional experience, eight 6–10 years, and seven had > 10 years of professional experience. The demographics of the final participants are shown in Table 1.

**Table 1:** Demographics of the final cohort

Variable	Number
**Age (in years)**	
25–35	8 (40)
36–45	10 (50)
> 45	2 (10)
**Position**	
Intern	6 (30)
Attending	1 (5)
Deputy senior physician	4 (20)
Senior physician	7 (35)
Chief physician	2 (10)
**Professional experience (in years)**	
≤ 5	5 (25)
6–10	8 (40)
> 10	7 (35)
**Gender**	
Male	20 (100)
Female	0 (0)
**Interest in technology**	
None	0 (0)
Medium	7 (35)
Very	13 (65)
**Area of expertise**	
Cardiology	5 (25)
OMFS	5 (25)
Orthopedic surgery	5 (25)
Radiology	5 (25)

Data: Values are given as absolute numbers (with percentages in brackets). OMFS = Oral and maxillofacial surgery

### Cases

**Fig. 1:**
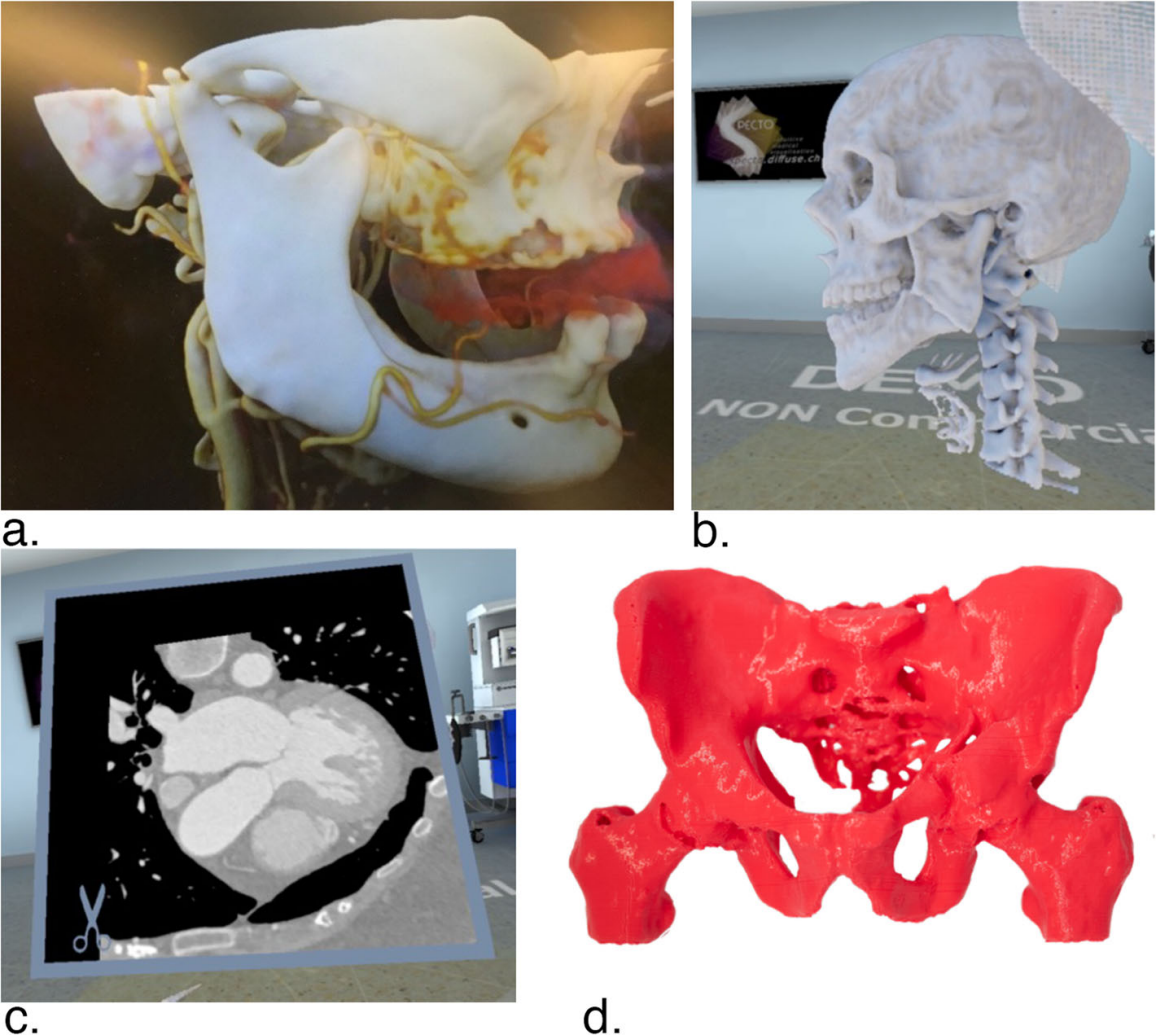
*Examples of prepared cases.* a. Dislocated right condylar fracture of the mandible *on 3D-Display (picture taken through passive shutter glasses).* b. Angle mandibular fracture *on VR-Glasses (corresponding two-dimensional view on computer-display).* c. Proximal crossing of RIVA and RCX *on VR-Glasses, CT View (corresponding two-dimensional view on computer-display).* d. Posterior column fracture left, 3D-printed. Data: RIVA = Ramus interventricularis anterior, RCX = Ramus circumflexus

Cases were chosen from a radiologist’s daily work at the University Hospital of Basel. Pathologies needed to be well recognized with the aforementioned 3D presentation systems while being similar to allow comparability. Furthermore, the chosen cases were required to be regularly occurring pathologies. In each discipline, except radiology, three different discipline-specific cases were selected to minimize bias. Each case was produced using all three modalities. In radiology, one case from each of the three other disciplines was used. In Table 2*,* the nine different cases are described. The preparation of all cases presented to the participants started with exporting DICOM (Digital Imaging and Communications in Medicine) data of the CT scans from the internal university hospital servers mentioned in Table 2. After that, the preparation of each of the three modalities proceeded differently. Figure 1 shows examples of prepared cases.

**Table 2:** Prepared cases

Area of expertise	Case 1	Case 2	Case 3
Cardiology	Proximal crossing of RIVA and RCX	Atypical retroaortal course of RCX with origin in the left coronary sinus	Atypical anteaortal course of left main trunk with common origin with RCA in the right coronary sinus
Oral and maxillofacial surgery	Dislocated right condylar fracture of the mandible	Mental mandibular fracture	Angle mandibular fracture
Orthopedic surgery	Transverse left acetabular fracture with contralateral pubic ramus fracture	Anterior column acetabular fracture with contralateral pubic ramus fracture	Posterior column fracture left

Data: RIVA = Ramus interventricularis anterior, RCX = Ramus circumflexus, RCA = Arteria coronaria dextra

### 3D-print

Exported DICOM data was segmented with 3D-Slicer (slicer.org, Version 4.10.0) [40] and final processing and integrity check was performed on Meshmixer (Autodesk, Inc., San Rafael, USA, Version 3.5.474). Slicing and printing were prepared with MakerBot Print (MakerBot Industries, New York City, USA, Version 4.6.0). Models were printed on a MEX MakerBot Replicator+ Printer (MakerBot Industries, New York City, USA) with a nozzle size of 0.4 mm, a slice thickness of 0.2 mm, and the printing material polylactic acid (PLA). Most cases were printed in 1:1 scale. However, the three orthopedic cases were printed in 0.5:1 scale due to the high print time and tall build volume needed for the 1:1 model. The time required to prepare varied between half an hour and one hour. Print duration reached from four to fifteen hours.

### VR-glasses

To prepare DICOM data for VR representation specto (Diffuse Inc., Basel, Switzerland, Version 3.3.0) was used. This software uses real-time GPU volume raytracing. A three-dimensional view was created through auto-segmentation, cutting for the region of interest, and an equivalent volume rendering color look-up table (LUT). Specto then uses the Steam VR platform (Steam, Valve Corporation, Bellevue, USA, Version 1.11.11) and HTC Vive VR-Glasses (High Tech Computer Corporation, Taoyuan, Taiwan) with a resolution of 1080 × 1200 pixels per eye for representation. Two controllers were used to interact with the virtual environment, including arbitrary cutting planes, individual lightning, and the possibility of switching to a CT view. The total time needed to prepare the cases varied between half an hour and an hour and a half.

### 3D-display

To prepare the cases for viewing on the 3D-Display, DICOM data was imported to Cinematic Rendering Prototype (Siemens Healthineers, Erlangen, Germany, Prototype), where different tools were enabled to crop the data on the region of interest and to apply specific LUT. The three-dimensional representation was then rendered and displayed with the Syngo VectorRayCaster (Siemens Healthineers, Erlangen, Germany, Prototype) on the 3D-Display HP Zvr Virtual Reality Display (HP Inc., Palo Alto, USA, no longer in production) with a resolution of 1920 × 1080 pixels. Similar to a 3D-TV, additional glasses (passive, polarized) had to be worn. The time needed from DICOM to final representation lasted between thirty minutes and one hour.

### Interviews & questionnaire

With nine cases available on all three modalities, interviews with the physicians were conducted by a 4th-year medical student. Each physician had an individual timeslot and was given a short introduction regarding methodology and the aim of the survey. The chronological order of the demonstrations followed a decreasing complexity concerning technical aspects. This approach was chosen in order to minimize the time physicians would spend in the lab in case of malfunctioning. Starting with the VR-Glasses, the participant was then presented with the first case from his discipline, followed by the presentation of the second case on the 3D-Display and a 3D-Model of the last case. While the physician was using each method, he gave oral feedback, which was written down. In the end, the physician filled out the missing information by pen. The rating scale ranged from 0 (very poor) to 10 (excellent) for the first research aim, i.e., understanding of the pathology, accuracy of details, quality of the anatomical representation, and technical operability. The accuracy of details evaluates how well details are represented e.g., the coronary arteries in the cardiology cases or the fracture gap in the maxillofacial surgery and orthopedic models. Technical operability was only assessed for VR-Glasses and 3D-Display as it concerned the user’s handling of the device used in the presentation i.e., the controllers and the mouse. Concerning the possible change in treatment, the participants were asked if in general they could imagine that the used method might lead to a change in the treatment plan. The original treatment plan was not provided, and participants were not familiar with the chosen cases. Furthermore, demographics were assessed, i.e., age, gender, discipline, years of professional experience, position within the hospital, and interest in technology. This entire procedure took around twenty minutes. For each of the disciplines the individual cases were shown. Radiologists received one case from each discipline. Cases within the discipline were randomly assigned to the participants using an alternating approach.

### Statistical methods

The data acquired with the paper and pencil questionnaires was transferred to and statistically analyzed with Microsoft Excel (Microsoft Corporation, Redmond, USA, Version 16.47.1). Descriptive statistics were used for an overview of participants’ characteristics. Mean, and standard deviation (SD) were reported for normally distributed data. Absolute numbers and percentages were given for binary and categorical variables.

### Ethics

A review by the institutional review board was waived. All patient data were anonymized. Furthermore, patients included in this study had already, by the time of the inclusion been treated. Therefore, this study had no impact on patients’ therapy or health.

## Results

### Averages

The averages including the radiologists’ feedback showed only marginal differences compared to the averages excluding the radiologists. Table 3 outlines the two averages.

**Table 3:** Comparison of the averages including the radiologists’ feedback with the average excluding the radiologists’ feedback

Variable	Average including radiologists’ feedback (*n* = 20)	Average excluding radiologists’ feedback (*n* = 15)
**Understanding of the pathology**		
3D-Print	7.5 (2.3)	7.6 (2.4)
VR-Glasses	8.1 (2.0)	8.0 (2.3)
3D-Display	7.6 (1.9)	7.6 (2.0)
**Accuracy of details**		
3D-Print	6.6 (2.3)	6.7 (2.1)
VR-Glasses	7.9 (1.5)	8.0 (1.4)
3D-Display	8.5 (1.5)	8.5 (1.6)
**Quality of the anatomical representation**		
3D-Print	7.6 (2.0)	7.7 (2.0)
VR-Glasses	8.4 (1.6)	8.4 (1.3)
3D-Display	8.3 (1.2)	8.3 (1.2)
**Technical operability**		
VR-Glasses	7.7 (1.8)	7.4 (1.8)
3D-Display	8.6 (1.6)	8.4 (1.8)

Data: The rating scale ranged from 0 (very poor) to 10 (excellent). Values are given as mean (with standard deviation (SD) in brackets)

### Disciplines and levels of professional experience

Understanding of the pathology: VR-Glasses are rated best in three out of four disciplines and two out of three levels of professional experience with an average of 8.1 (SD 2.0) followed by 3D-Display with 7.6 (SD 1.9) and 3D-Print with 7.5 (SD 2.3).

Accuracy of details: 3D-Display is rated best in three out of four disciplines and in all levels of professional experience. 3D-Print is last in all disciplines and all levels of professional experience. Mean of 3D-Display is 8.5 (SD 1.5), VR-Glasses 7.9 (SD 1.5) and, 3D-Print 6.6 (SD 2.3).

Quality of the anatomical representation: VR-Glasses have the best score with 8.4 (SD 1.6), closely followed by 3D-Display with 8.3 (SD 1.2) and with some offset 3D-Print with 7.6 (SD 2.0). VR-Glasses reach the best score in three out of four disciplines and two out of three levels of professional experience.

Technical operability: 3D-Display is consistently rated best in all levels of professional experience and all disciplines with an average of 8.6 (SD 1.6). VR-Glasses rated second with 7.7 (SD 1.8). 3D-Print was not assessed.

Comparing all three methods and all research questions above, the cardiologists gave the lowest rating in all queries and modalities except in one. On the other hand, orthopedic surgeons gave the best ratings except in one case. Tables 4 and 5 show all scores stratified in disciplines and level of professional experience.

**Table 4:** Comparison of 3D-Print, VR-Glasses and 3D-Display concerning disciplines

Variable	Cardiology (*n* = 5)	OMFS (*n* = 5)	Ortho (*n* = 5)	Radiology (n = 5)
**Understanding of the pathology**				
3D-Print	5.8 (2.5)	8.2 (1.9)	8.8 (1.5)	7.2 (2.1)
VR-Glasses	7.2 (1.7)	9.0 (1.5)	7.8 (3.0)	8.2 (0.4)
3D-Display	7.0 (2.2)	6.6 (1.4)	9.2 (1.2)	7.6 (1.4)
**Accuracy of details**				
3D-Print	4.8 (1.7)	7.4 (0.8)	8.0 (2.1)	6.2 (2.6)
VR-Glasses	7.6 (1.5)	8.0 (1.1)	8.4 (1.5)	7.6 (1.9)
3D-Display	7.4 (1.7)	8.6 (1.0)	9.4 (1.2)	8.6 (1.2)
**Quality of the anatomical representation**				
3D-Print	6.4 (2.4)	8.4 (1.0)	8.4 (1.6)	7.0 (1.8)
VR-Glasses	7.6 (0.8)	8.6 (1.0)	9.0 (1.5)	8.2 (2.1)
3D-Display	7.8 (1.3)	8.2 (0.4)	8.8 (1.5)	8.2 (1.2)
**Technical operability**				
VR-Glasses	6.0 (1.5)	7.6 (1.4)	9.0 (1.2)	8.6 (1.2)
3D-Display	7.8 (2.0)	7.8 (1.5)	9.8 (0.4)	9.4 (0.5)

Data: The rating scale ranged from 0 (very poor) to 10 (excellent). Values are given as mean (with standard deviation (SD) in brackets). OMFS = Oral and maxillofacial surgery, Ortho = Orthopedic surgery

**Table 5:** Comparison of 3D-Print, VR-Glasses and 3D-Display concerning professional experience (in years)

Variable	≤5 years (*n* = 5)	6–10 years (*n* = 8)	> 10 years (*n* = 7)
**Understanding of the pathology**			
3D-Print	8.2 (1.5)	7.0 (2.7)	7.6 (2.3)
VR-Glasses	8.6 (1.5)	8.3 (0.8)	7.4 (2.9)
3D-Display	8.0 (1.9)	7.4 (1.7)	7.6 (1.9)
**Accuracy of details**			
3D-Print	7.0 (0.9)	5.5 (2.4)	7.6 (2.2)
VR-Glasses	7.2 (1.9)	8.0 (0.9)	8.3 (1.7)
3D-Display	9.2 (0.7)	8.1 (1.9)	8.4 (1.2)
**Quality of the anatomical representation**			
3D-Print	7.6 (1.0)	6.4 (2.2)	8.9 (1.4)
VR-Glasses	7.0 (1.7)	8.6 (1.3)	9.0 (1.1)
3D-Display	9.0 (0.9)	7.9 (1.1)	8.1 (1.4)
**Technical operability**			
VR-Glasses	8.0 (1.7)	7.5 (1.5)	7.8 (2.1)
3D-Display	8.6 (1.4)	9.1 (0.6)	8.0 (2.3)

Data: The rating scale ranged from 0 (very poor) to 10 (excellent). Values are given as mean (with standard deviation (SD) in brackets)

### Change in treatment

In cardiology, two out of three participants stated a possible change in treatment when using the VR-Glasses. Furthermore, three out of five radiologists reported a possible change in treatment when using VR-Glasses. In the group with a professional experience of more than > 10 years, no change in treatment could be envisioned for any of the three methods. Table 6 shows the percentage of participants who stated a change in treatment is possible using a 3D-Print, VR-Glasses, or a 3D-Display.

**Table 6:** Possible change in treatment, a. disciplines, b. professional work experience (in years)

		Disciplines					Years of experience			
	Average (*n* = 18)	a.	Cardiology (*n* = 3)	OMFS (*n* = 5)	Ortho (*n* = 5)	Radiology (*n* = 5)	b.	≤ 5 (*n* = 5)	6–10 (*n* = 7)	> 10 (*n* = 6)
3D-Print	6 (33)		0	2 (40)	2 (40)	2 (40)		2 (40)	4 (57)	0
VR-Glasses	8 (44)		2 (67)	1 (20)	2 (40)	3 (60)		3 (60)	5 (71)	0
3D-Display	6 (33)		1 (33)	1 (20)	2 (40)	2 (40)		2 (40)	4 (57)	0

Data: Values are given as absolute numbers (with percentages in brackets) for binary and categorical variables. OMFS = Oral and maxillofacial surgery, Ortho = Orthopedic surgery

## Discussion

Medical imaging such as computed tomography and magnetic resonance imaging plays an essential role in developing a diagnosis. These medical image datasets are acquired in three dimensions, yet they are mostly presented on a two-dimensional display. The present study was designed to compare different three-dimensional representation methods, namely 3D-Print, VR-Glasses, and 3D-Display to understand these methods better.

All three modalities were well accepted and received a high rating in understanding of the pathology, accuracy of details, quality of the anatomical representation, and operability. Furthermore, a possible change in treatment was stated using 3D-Print or 3D-Display in one-third of the cases. Using the VR-Glasses, an even higher number of physicians (44%) reported a possible change in treatment. However, this is only true in participants with less than ten years of experience. This could indicate a possible educational component that should be explored further.

Our conclusions accord to some extent with previous studies which revealed significant potential for 3D-Printing [3, 41] and VR-Glasses [22, 25]. A study by Bartella, Kamal, Scholl, et al. evaluated the use of VR-Glasses in preoperative planning in maxillofacial surgery. They presented three different cases to four participants, which had to rate them concerning, e.g., the potential for clinical use. The authors have noted the importance of VR-Glasses in preoperative planning and teaching [42]. However, the participants were only given a chance to evaluate VR-Glasses. The strength of this study is that three different methods of 3D representations were compared. Some new conclusions can therefore be drawn. First, all methods are very well accepted by physicians. Consequently, further evaluations of the function and characteristics of these modalities to gain a more profound knowledge of their use in medicine is warranted. Second, it is somewhat surprising that the cardiologist gave the lowest rating in all questions and modalities in comparing the three methods except in one. On the other hand, orthopedic surgeons gave the best ratings except in one case. A possible explanation for this might be that gross bone fractures were chosen in all three orthopedic cases. In contrast, cardiology included three delicate coronary pathologies. This might indicate a weakness of all three methods in presenting delicate, detailed structures. This holds especially true for the cinematic rendering approach of VR and 3D-Display were elaborate segmentation technology as for 3D-Printing was not available and as such could have been obscured by neighboring structures with similar voxel intensities. This is particularly the case in cardiology. Furthermore, cardiologists are generally used to angiographies while the other disciplines routinely use CT data. This might make it more difficult for cardiologists to interact with the 3D representations.

Third, many physicians with professional work experience of less than ten years stated a possible change in treatment. However, physicians with professional experience above ten years reported in no case a possible change in therapy. This result is, to some extent, not surprising. The more experienced, the less someone can benefit from new methods. Nevertheless, this result was not expected to be so clear. This, however, renders the idea of using these three-dimensional methods as teaching tools for less experienced physicians [20, 22, 30, 42]. Lastly, the averages including the radiologists’ feedback and the averages excluding their feedback differed only slightly. This could indicate a good overall acceptance by physicians who are not familiar with these new methods. Only the averages concerning technical operability were slightly higher when including the radiologists. This could be because radiologists at the University Hospital of Basel occasionally work with VR-Glasses and 3D-Displays.

The cases chosen were regularly occurring pathologies, often with a standardized treatment plan. In choosing challenging and unusual cases, a possible change in treatment could have been stated more often. This is because the optimal therapy is more difficult to derive in complex cases. The strength of 3D representations is the presentation of challenging cases. This could have had an impact especially in the group with more than ten years of work experience.

Owing to some limitations in the sampling process, in choosing and preparing the cases and technological aspects, our findings should be interpreted with caution. The main limitation is the relatively small sample size. It proved to be very difficult to recruit a sufficient number of participants. Of fifty-two possible participants contacted, only twenty-three responded, some of them only after being contacted several times. The sample size consists of twenty male physicians selected from one university hospital. These numbers could, in further studies, be increased and diversified. However, as physicians are often hardly available due to the high workload, it is challenging to acquire more participants. This could be met in expanding the range of included hospitals and transferring the test site closer to the workplace of targeted participants. Furthermore, there was some element of selection bias. Predominantly, physicians interested in new technology were more likely to self-select to participate in this study.

Cases selected were considered suitable for three-dimensional representation and from a radiologist’s daily repertoire. Therefore, these cases represent only some possibilities. Additionally, specific fields were chosen, i.e., coronary pathologies in cardiology and bone-fractures in orthopedics and oral and maxillofacial surgery. This does not represent the entire range of eventualities. It would have been interesting to place the focus on challenging and unusual cases as this is the main strength of 3D representations. However, rather general cases were chosen so the educational value in the training of young and inexperienced physicians could also be assessed. The number of cases participants tested on each modality could also be increased given that only one case per modality was shown. It would be interesting to determine whether the groups with different professional experience react differently to distinct complexities of cases. The number of three cases per specific field was not suitable as some case/modality combinations were seen more often than others. The fields chosen could also have been more diverse e.g., neurological, or abdominal pathologies would have increased the meaningfulness of this study. Lastly, by first presenting the pathology with the VR-Glasses, bias could have been introduced. However, this was minimized as cases were switched between the different methods, and participants were not informed as to why VR-Glasses were used first.

With regards to the research question concerning accuracy of details, no comparison was given such as a CT-View. This question was based on the participants clinical experience. Most physicians are familiar with interpreting CT and MRI datasets or e.g., cardiologists with a coronary angiography. This allowed the participants to compare the three-dimensional presentations with the images they were already acquainted with. Concerning the research question for possible change in treatment, the answers were based on the respective physicians’ clinical experience and their subjective decision-making. This is often the case in the day-to-day work of physicians when deciding on the personal treatment plan. An original treatment plan was not provided as this could have led to a response bias.

Technical limitations appear when rendering or segmenting the raw DICOM data. This step has a specific susceptibility to errors, and the procedure must be conducted with caution and high accuracy. Furthermore, the technical equipment showed some shortcomings. The models were printed using the MEX technology as it was widely available in the used lab. A study by Msallem et al. attested the MEX technology the highest overall precision compared to other printing technologies [43]. The layer height of 0.2 mm was rather thick and could be lowered in further studies. However, considering the chosen cases, the pathologies were adequately represented with the employed printing resolution. Any possible negative effects regarding the haptic experience were considered to be minor. The orthopedics models were printed in a 0.5:1 scale due to the high print time and tall build volume needed. This led to a decrease in details and haptic recognition. This was noted by several orthopedic surgeons who mentioned that if the models had been 1:1, a higher rating would have been achieved. The HTC Vive VR-Glasses (High Tech Computer Corporation, Taoyuan, Taiwan) that were used have a screen resolution of 1080 × 1200 pixels per eye. Newer versions of VR-Glasses hold much higher resolutions, leading to more precise representations of pathologies. This is a significant improvement that has to be accounted for in interpreting the results of this study. Moreover, the 3D-Display that was used is no longer being produced. The software used to prepare the 3D-Prints were not CE marked medical software. This is also true for the software used to prepare the DICOM data for VR representation and for representation on the 3D-Display. However, a CE marking is not thought to be necessary as this study was conducted within an experimental research context. Furthermore, the CE label does not implicate quality and is not a certification mark. Finally, some technical difficulties occurred concerning the VR-Glasses. This included and was not limited to loosen contact and the inability to render some DICOM data. This problem must be addressed technically.

## Conclusions

3D-Print, VR-Glasses, and 3D-Displays are very well accepted, and a relevant percentage of physicians with less than ten years of professional work experience could imagine a possible change in treatment using any of these three-dimensional methods. These findings have important implications for further development of three-dimensional representation tools, given their high acceptance and, in our understanding, a high percentage of physicians stating a possible change in treatment. Our findings challenge scientists, technicians, and physicians to further develop these three-dimensional representation methods to improve the three-dimensional understanding of pathologies. Furthermore, possible applications in medicine must be evaluated more clearly. On the one hand, they can facilitate the day-to-day work of physicians. On the other hand, the educational value could be of importance in the training of young and inexperienced physicians.

## Data Availability

All data generated or analysed during this study are included in this published article.

## Abbreviations

CT: Computed tomography
MRI: Magnetic resonance imaging
VR: Virtual Reality
MEX: Material extrusion
VP: Vat photopolymerization
PLA: Polylactic acid
ABS: Acrylonitrile butadiene styrene
DICOM: Digital Imaging and Communications in Medicine
LUT: Look-up-Table
SD: Standard deviation
